# Clinicopathological characteristics and prognostic factors of cervical adenocarcinoma

**DOI:** 10.1038/s41598-021-86786-y

**Published:** 2021-04-05

**Authors:** Min Wang, Bo Yuan, Zhen-huan Zhou, Wei-wei Han

**Affiliations:** 1 Key Laboratory for Molecular Enzymology and Engineering of Ministry of Education, Engineering Laboratory for AIDS Vaccine, School of Life Science, Jilin University, 2699 Qianjin Street, Changchun, 130012 China; 2 Department of Urology, The First Hospital of Jilin University, Changchun, 130021 China; 3 Reproductive Medical Center, The Second Hospital of Jilin University, Changchun, 130041 China

**Keywords:** Cancer, Oncology

## Abstract

We aimed to assess the clinicopathological features and to determine the prognostic factors of cervical adenocarcinoma (AC). Relevant data were extracted from surveillance, epidemiology and end results database from 2004 to 2015. The log-rank test and Cox proportional hazard analysis were subsequently utilized to identify independent prognostic factors. A total of 3102 patients were identified. The enrolled patients were characterized by higher proportion of early FIGO stage (stage I: 65.9%; stage II: 14.1%), low pathological grade (grade I/II: 49.1%) and tumor size ≤ 4 cm (46.8%). The 5- and 10-year cancer-specific survival rates of these patients were 74.47% and 70.00%, respectively. Meanwhile, the 5- and 10-year overall survival (OS) rates were 71.52% and 65.17%, respectively. Multivariate analysis revealed that married status, surgery as well as chemotherapy were independent favorable prognostic indicators. Additionally, aged > 45, tumor grade III/IV, tumor size > 4 cm, advanced FIGO stage and pelvic lymph node metastasis (LNM) were unfavorable prognostic factors (all *P* < 0.01). Stratified analysis found that patients without surgery could significantly benefit from chemotherapy and radiotherapy. In addition, chemotherapy could significantly improve the survival in stage II–IV patients and radiotherapy could only improve the survival in stage III patients (all *P* < 0.01). Marital status, age, grade, tumor size, FIGO stage, surgery, pelvic LNM and chemotherapy were significantly associated with the prognosis of cervical AC.

## Introduction

Uterine cervix carcinoma is a threatening cause of cancer-related death in females, which is reported to have approximately 311,000 death cases and 570,000 new cases in 2018^1^. Squamous cell carcinoma (SCC) is the most prevalent histological type of cervical cancer and approximately 10–25% of cervical cancer is adenocarcinoma (AC)^2, 3^. Additionally, the prevalence of cervical AC has been reported to increase in multiple regions^4^, the proportion of which has been demonstrated to double in the last decade^5^. However, the knowledge of cervical AC is currently limited to small case series, with unclear clinicopathological features and standard treatment^6, 7^.

The standard therapeutic regimen of cervical AC is currently the same as SCC, which includes radical hysterectomy along with adjuvant radiotherapy (RT), radical hysterectomy or primary RT for early-stage cancer. In addition, concurrent chemoradiotherapy (CCRT) is prevalently recommended and promoted for locally advancedcancer as well as early-stage FIGO lesions^8^, which gives rise to equivalent outcomes. Nevertheless, cervical SCC and AC patients even with the same Federation International of Gynecology and Obstetrics (FIGO) stage still have disparate prognostic outcomes^4, 9, 10^. At present, whether the standard therapeutic regimen is equally suitable for SCC and AC patients has been doubted due to poorer prognostic outcomes of AC patients than SCC^4, 10^. Therefore, in order to provide a better theoretical therapeutic basis for cervical AC, it is necessary to further understand the survival and prognosis of cervical AC patients. Although some previous studies have demonstrated that FIGO stage^11–13^, nodal status^11, 12^, tumor size^11, 13^, age and tumor grade^12, 14^ were prognostic factors of cervical AC, however, the numbers of patients enrolled in these studies were small, and with inconsistent results. Therefore, it is significant to examine the prognostic indicators for cervical AC based on a large population, aiming at establishing a framework for new therapeutic strategies.

The NCI-supported Surveillance, Epidemiology and End Results (SEER) database, the most authoritative and largest cancer dataset in North America^15^, reports tumor data on approximately 30% of the US population by selecting relevant registries to represent population diversity^16^. Therefore, SEER is a valuable database to investigate such rare tumors^17, 18^. Therefore, a retrospective study was conducted by collecting eligible patients from SEER database, aiming at summarizing clinical features, survival and treatment for patients with cervical AC to delineate prognostic factors.

## Materials and methods

### Ethics statement

It was a population-based retrospective study using SEER database. To acquire relevant data from the database, we signed the SEER Research Data Agreement (No.19817-Nov2018) and further searched for data based on the approved guidelines. All extracted data were publicly accessible and de-identified, and data analysis was considered to be non-human subjects by Office for Human Research Protection. Thus, no approval was requested by the institutional review board.

### Study population

SEER*State v8.3.6 (released on August 8, 2019) was utilized to select and identify qualified subjects, which includes 18 SEER regions from 2004 to 2015 (2018 submission). The inclusion criteria were as follows: (1) primary cervical AC patients; (2) the diagnosis of cervical AC was based on ICD-O-3; coded as 8140–8490^19, 20^. Patients were eliminated if they had: (1) more than one primary malignancies; (2)reported diagnosis source from autopsy or death certificate or without pathological diagnosis; (3)without certain necessary clinicopathological data, including surgical style as well as FIGO stage; (4) without prognostic information. The rest of subjects were enrolled as the initial cohort of SEER.

### Covariates and endpoint

The following clinicopathological parameters were analyzed: year of diagnosis (2004–2007, 2008–2011, 2012–2015)^21^; marital status (unmarried, married);race (black, whiteor others);insured status (uninsured/unknown, any medicaid/insured); age(≤ 45, > 45); grade (grade I/II, grade III/IV, unknown); FIGO stage (stage I, II, III, IV); tumor size (≤ 4 cm, > 4 cm, unknown); pelvic lymph node (LN) dissections (none or biopsy, removal of 1 to 3 regional LNs, removal of ≥ 4 regional LNs), pelvic lymph node metastasis (LNM) (positive, negative and unknown);surgery (no surgery, local tumor excision, total hysterectomy), chemotherapy (no/unknown, yes) and radiotherapy(no/unknown, yes). Patients with widowed or single (never married or having a domestic partner) or divorced or separated status were all classified as unmarried^22, 23^. All of the eligible cases were re-identified according to the 2018 FIGO staging criteria^24, 25^. Median age at diagnosis was 45 years old in our study, which was used as the cutoff value for age classification. Meanwhile, the classification of tumor size and age was also based on previous researches^6, 26^. CCRT was defined as the addition of chemotherapy during radiotherapy. Definitive radiotherapy indicated that only radiotherapy was used in the treatment^27^. The endpoints of our research included overall survival (OS) and cancer‐specific survival (CSS). The former was defined as the duration from diagnosis to all-cause death, and the latter referred to the duration from diagnosis to cervical AC-caused death.

### Statistical analyses

Kaplan–Meier (K–M) method was employed to estimate the univariate analysis, followed by log-rank test for assessing the differences of CSS and OS among different groups. Variables with *P* values ≤ 0.1 in the univariate analysis were further incorporated into the multivariate Cox proportional hazard analysis. In addition, stratified analysis was performed by using Cox regression analysis. SPSS software (SPSS Inc., Chicago, USA, version 19.0) was utilized for statistical analysis, and GraphPad Prism 5 was utilized for plotting survival curves. These softwares have recieved permission and freely available. A two-sided *P* < 0.05 was considered as statistically significant. These softwares have been approved.

## Results

### Patients’ characteristics

**Figure 1:**
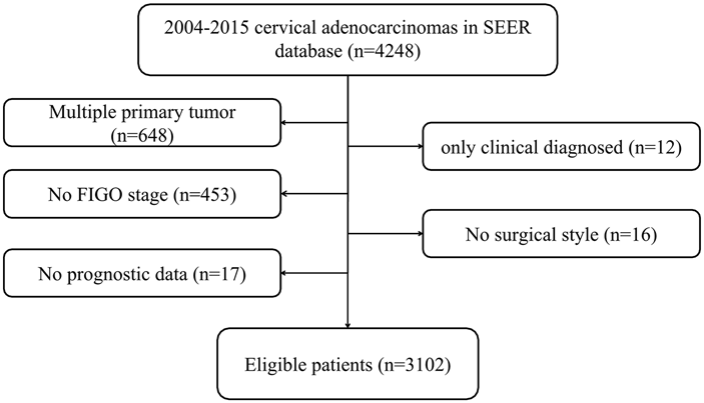
Flow chart of patient screening.

A total of 3102 cervical AC patients were identified, including 2044 (65.9%) patients with stage I, 437 (14.1%) patients with stage II, 510 (16.4%) patients with stage III and 111 (3.6%) patients with stage IV. The detailed screening process was shown in Fig. 1. Patient features and therapeutic regimens were listed in Table 1. To be specific, the median age was 45 years (range 6–98 years). Among them, 11 cases (0.4%) were ≤ 18 years old, 1618 (52.20%) were ≤ 45 years old, and 422 cases (13.6%) were ≥ 65 years old. Most of cervical AC cases were of low pathological grade (grade I/II: 49.1%), had tumor size ≤ 4 cm (46.8%) and were treated by surgery (69.4%). More patients received ≥ 4 pelvic LN dissection (47.6%) and 12.6% of them had positive pelvic LN.

**Table 1:** The clinicopathological characteristics and treatment of the included 3102 cervical adenocarcinomas patients

Variable	N (%)
**Year at diagnosis**	
2004–2007	893 (28.8%)
2008–2011	1062 (34.2%)
2012–2015	1147 (37.0%)
**Insured status**	
Uninsured/unknown	838 (27.0%)
Any medicaid/insured	2264 (73.0%)
**Insured stutus**	
Unmarried	1512 (48.7%)
Married	1590 (51.3%)
**Age**	
≤ 45	1618 (52.2%)
> 45	1484 (47.8%)
**Race**	
Black	237 (7.6%)
White	2493 (80.4%)
Other	372 (12.0%)
**Grade**	
Grade I/II	1524 (49.1%)
Grade III/IV	769 (24.8%)
Unknown	809 (26.1%)
**FIGO stage**	
Stage I	2044 (65.9%)
Stage II	437 (14.1%)
Stage III	510 (16.4%)
Stage IV	111 (3.6%)
**Tumor size**	
≤ 4 cm	1453 (46.8%)
> 4 cm	722 (23.3%)
Unknown	927 (29.9%)
**Surgery**	
No surgery	948 (30.6%)
Local tumor excision	367 (11.8%)
Total hysterectomy	1787 (57.6%)
**Lymph node dissection**	
None or biopsy	1553 (50.1%)
1–3	72 (2.3%)
≥ 4	1477 (47.6%)
**Pelvic lymph node metastasis**	
Negative	1407 (45.4%)
Positive	206 (6.6%)
Unknown	1489 (48.0%)
**Chemotheray**	
No/unknown	1968 (63.4%)
Yes	1134 (36.6%)
**Radiotherapy**	
No/unknown	1845 (59.5%)
Yes	1257 (40.5%)

### Patient survival

**Figure 2:**
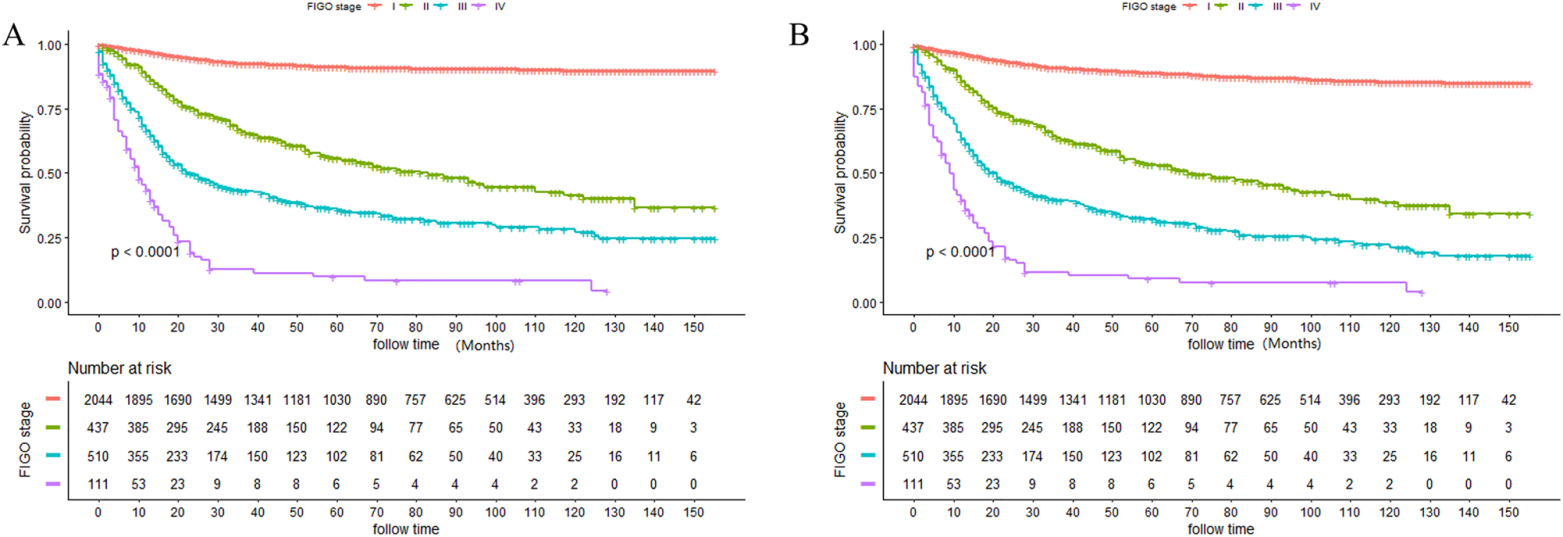
Kaplan–Meier curves stratified by FIGO stage of CSS (**A**) and OS (**B**).

The median survival was 45.0 months. The 3-, 5- and 10-year CSS rates were 77.97%, 74.47% and 70.00%, respectively. Meanwhile, the 3-, 5- and 10-year OS rates were 75.56%,71.52% and 65.17%, respectively. K–M curves stratified by FIGO stage were displayed in Fig. 2A (CSS) and Fig. 2B (OS). Notably, patients with stage III and IV had significantly poorer prognosis than those with stage I and II (*P* < 0.0001 for both). Moreover, the 5-year CSS and OS rates for patients were stage I: 90.43% and 88.08%; stage II: 55.53% and 53.19%; stage III: 23.95% and 20.45%; and stage IV: 9.77% and 8.90%. In addition, the 2-year, 5-year and 10-year survival rates of patients with different tumor grades were listed in Table 2.

**Table 2:** The 2-year, 5-year and 10-year survival rates of patients with different tumor grades.

Grade	Number	Cancer‐specific survival			Overall survival		
		2-year (%)	5-year (%)	10-year (%)	2-year (%)	5-year (%)	10-year (%)
Grade I	694	95.1	91.5	89.9	94.4	89.8	86.4
Grade II	830	89.1	81.8	76.3	87.7	79.1	71.9
Grade III	664	61.4	50.7	44.3	58.8	47.7	39.8
Grade IV	105	58.7	48.8	48.8	55.9	42.4	35.6

### Prognostic factors for survival

Univariate analysis revealed that insured status, marital status, age, race, grade, tumor size, FIGO stage, surgery, number of pelvic LN dissections, pelvic LNM, chemotherapy and radiotherapy were prognostic indicators for CSS and OS (all *P * < 0.05). Multivariate analysis revealed that married (HR: 0.769, 95% CI 0.662–0.894, *P* < 0.001) and surgery [(local tumor excision) HR: 0.568, 95%CI: 0.421–0.766, *P* < 0.001; (total hysterectomy) HR: 0.439, 95% CI 0.336–0.576, *P* < 0.001] were independent favorable prognostic factors of CSS. However, age > 45 (HR: 1.631, 95% CI 1.364–1.950, *P* < 0.001). grade III/IV (HR: 2.116, 95% CI 1.761–2.541, *P* < 0.001), tumor size > 4 cm (HR: 1.628, 95% CI 1.292–2.051, *P* < 0.001) and advanced FIGO stage (*P* < 0.001) were independent unfavorable prognostic indicators of CSS. The results of multivariate analysis in OS were similar to those of in CSS. Besides, pelvic LNM (HR: 1.648, 95% CI 1.196–2.271, *P* = 0.002) and chemotherapy (HR: 0.685, 95% CI 0.567–0.827, *P* < 0.001) were also independent prognostic factors for OS (Table 3).

**Table 3:** Univariate and multivariate analyses of cancer special survival (CSS) and overall survival (OS) for patients.

Variables	CSS			OS		
	Univariate analysis	Multivariate analysis		Univariate analysis	Multivariate analysis	
	*P *value	HR (95% CI)	*P *value	*P *value	HR (95% CI)	*P *value
**Year at diagnosis**	0.788		NI	0.591		NI
2004–2007						
2008–2011						
2012–2015						
**Insured stutus**	0.063		0.151	0.033		0.902
Uninsured/unknown		Reference			Reference	
Any medicaid/insured		0.891 (0.761, 1.043)			0.902 (0.780, 1.044)	
**Marital status**	< 0.001		0.001	< 0.001		< 0.001
Unmarried		Reference			Reference	
Married		0.769 (0.662, 0.894)			0.752 (0.654, 0.865)	
**Age**	< 0.001		< 0.001	< 0.001		< 0.001
≤ 45		Reference			Reference	
> 45		1.631 (1.364, 1.950)			2.027 (1.709, 2.405)	
**Race**	< 0.001		0.383	< 0.001		0.158
Black		Reference			Reference	
White		0.858 (0.692, 1.065)	0.858		0.824 (0.676, 1.004)	0.055
Other		0.380 (0.658, 1.173)	0.878		0.842 (0.644, 1.101)	0.208
**Grade**	< 0.001		< 0.001	< 0.001		< 0.001
Grade I/II		Reference			Reference	
Grade III/IV		2.116 (1.761, 2.541)	< 0.001		2.066 (1.743, 2.448)	< 0.001
Unknown		1.179 (0.961, 1.446)	0.115		1.189 (0.987, 1.433)	0.069
**FIGO stage**	< 0.001		< 0.001	< 0.001		< 0.001
Stage I		Reference			Reference	
Stage II		2.679 (2.059, 3.486)	< 0.001		2.156 (1.698, 2.737)	< 0.001
Stage III		4.968 (3.843, 6.422)	< 0.001		4.039 (3.211, 5.080)	< 0.001
Stage IV		9.029 (6.645, 12.267)	< 0.001		6.918 (5.214, 9.178)	< 0.001
**Tumor size**	< 0.001		< 0.001	< 0.001		< 0.001
≤ 4 cm		Reference			Reference	
> 4 cm		1.628 (1.292, 2.051)	< 0.001		1.513 (1.227, 1.868)	< 0.001
Unknown		1.638 (1.306, 2.055)	< 0.001		1.546 (1.261, 1.894)	< 0.001
**Surgery**	< 0.001		< 0.001	< 0.001		< 0.001
No surgery		Reference			Reference	
Local tumor excision		0.568 (0.421, 0.766)	< 0.001		0.516 (0.389, 0.682)	< 0.001
Total hysterectomy		0.439 (0.336, 0.576)	< 0.001		0.370 (0.287, 0.477)	< 0.001
**Lymph node dissection**	< 0.001		0.190	< 0.001		0.441
None or biopsy		Reference			Reference	
1–3		1.092 (0.586, 2.035)	0.782		1.234 (0.678, 2.246)	0.491
≥ 4		0.742 (0.448, 1.229)	0.247		0.922 (0.570, 1.493)	0.743
**Pelvic lymph node metastasis**	< 0.001		0.063	< 0.001		0.005
Negative		Reference			Reference	
Positive		1.481 (1.044, 2.101)	0.028		1.648 (1.196, 2.271)	0.002
Unknown		1.503 (0.899, 2.514)	0.120		1.681 (1.030, 2.745)	0.038
**Chemotheray**	< 0.001		0.067	< 0.001		< 0.001
No/unknown		Reference			Reference	
Yes		0.823 (0.668, 1.014)			0.685 (0.567, 0.827)	
**Radiotherapy**	< 0.001		0.074	< 0.001		0.138
No/unknown		Reference			Reference	
Yes		0.827 (0.671, 1.019)			0.864 (0.712, 1.048)	

*CSS* cancer‐specific survival, *OS* overall survival, *NI* not included in the multivariate survival analysis.

### Stratified analysis of the effect of chemotherapy and radiotherapy on survival

To explore the benefits of chemotherapy and radiotherapy, we performed stratified analysis of patients with different FIGO stage and surgical style. As a result, patients with stage III/IV could significantly benefit from chemotherapy (both CSS and OS) (*P* < 0.001), and stage II patients could benefit in terms of OS (*P* = 0.004). Meanwhile, patients without surgery could also significantly benefit from chemotherapy and radiotherapy (*P* < 0.05). In addition, only patients with stage III could significantly benefit from radiotherapy (*P* < 0.001) (Tables 4, 5).

**Table 4:** Stratified analysis of cancer-specific survival (CSS) and overall survival (OS) for chemotherapy in different FIGO stage and surgery style.

Variables	CSS		OS	
	HR (95 CI)	*P *value	HR (95 CI)	*P* value
**FIGO stage**				
Stage I	1.49 (0.94, 2.37)	0.092	0.95 (0.65, 1.38)	0.790
Stage II	0.68 (0.43, 1.09)	0.107	0.54 (0.36, 0.82)	0.004
Stage III	0.59 (0.44, 0.79)	< 0.001	0.56 (0.43, 0.72)	< 0.001
Stage IV	0.31 (0.18, 0.52)	< 0.001	0.35 (0.22, 0.55)	< 0.001
**Surgery**				
No surgery	0.73 (0.58, 0.91)	0.006	0.62 (0.50, 0.76)	< 0.001
Local tumor excision	1.39 (0.62, 3.12)	0.430	0.99 (0.49, 2.01)	0.986
Total hysterectomy	4.23 (2.51, 7.11)	< 0.001	2.68 (1.69, 4.25)	< 0.001

Adjustment variables: marital status; age; grade; tumor size; pelvic lymph node metastasis; radiotherapy.

**Table 5:** Stratified analysis of cancer-specific survival (CSS) and overall survival (OS) for radiotherapy in different FIGO stage and surgery style.

Variables	CSS		OS	
	HR (95 CI)	*P *value	HR (95 CI)	*P *value
**FIGO stage**				
Stage I	1.40 (0.86, 2.28)	0.179	1.34 (0.90, 2.01)	0.147
Stage II	0.84 (0.50, 1.41)	0.504	0.88 (0.55, 1.43)	0.618
Stage III	0.47 (0.35, 0.62)	< 0.001	0.49 (0.38, 0.65)	< 0.001
Stage IV	0.74 (0.46, 1.18)	0.208	0.74 (0.47, 1.16)	0.191
**Surgery**				
No surgery	0.57 (0.45, 0.72)	< 0.001	0.60 (0.48, 0.74)	< 0.001
Local tumor excision	5.76 (1.98, 16.79)	0.001	5.11 (2.03, 12.83)	< 0.001
Total hysterectomy	1.27 (0.82, 1.93)	0.287	1.22 (0.82, 1.82)	0.332

## Discussion

This population-based study revealed the clinicopathological features as well as survival of patients with cervical AC. Cervical AC accounts for only approximately 20–25% of all cervical carcinomas^2, 3^. AC is the second most common type of primary cervical cancer, secondly only to SCC^28^. Previous studies predominantly enrolling patients with SCC have provided most of the present therapeutic knowledge on cervical cancer^29, 30^. However, the different outcomes of AC have been rarely reported. Furthermore, prospective studies have not solely focused on the treatment of AC. Consequently, our understanding of the natural history, prognosis factors and optimal management of cervical AC is limited ^31^. To this end, we aimed at describing the clinicopathological features and treatment, as well as examining prognostic indicators for cervical AC by including a total of 3102 cervical AC patients.

Previous studies have also explored the prognostic factors of cervical AC patients. The review of 222 surgically-treated cervical AC with stage Ia2-IIa disease by Park et al. found that nodal status and parametrial involvement were independent prognostic factors for disease-free survival (DFS) and OS^13^. In addition, the analysis of 46 patients with stage I-IV cervical AC revealed that FIGO stage was the only independent prognostic factor for both DFS and OS^11^. A retrospective Dutch study assessing 305 cases of cervical AC found that tumor size, tumor grade and LNM remained as significant independent predictors for survival^12^. Although most of these studies are small-size and single-center retrospective studies, with consistent results to ours. In addition, we also found that marital status is an independent prognostic factor for cervical AC.

The same therapeutic strategy is recommended for SCC and AC according to the present guidelines. Nevertheless, there have been no consistent data concerning the therapeutic efficacy in different histological classification^7^. Surgery and radiotherapy are recommended as the primary therapeutic regimes for early-stage cervical cancer in accordance with NCCN guidelines^8^. In addition, the 5-year OS rates for stage IA1 and stage IA2 lesions were 96.5% and 99.4%, respectively, for radical hysterectomy, 96.6% and 100%, respectively, for local excision, 98.4% and 96.9%, respectively, for simple hysterectomy in a study enrolling 1567 patients with cervical AC^32^. Our study also found that surgery is an independent favorable prognostic factor.

Radiotherapy is an alternative option for patients who are not suitable for surgery or who refuse surgery. For patients with stage IB2-IVA cervical cancer, concurrent cisplatin based-chemoradiotherapy plus brachytherapy was the standard therapeutic regimen^7^. Our study found that radiotherapy and chemotherapy could provide significant survival benefits among patients without surgery. However, in terms of tumor stage, only patients with stage III could gain significant survival benefits from radiotherapy. The worse efficacy of cervical AC is possibly caused by insensitivity of radiotherapy. Cervical AC patients have been reported to have poorer complete response (CR) as well as local control rates, therefore requiring longer time to obtain CR than SCC populations following CCRT or definitive radiotherapy^29, 33, 34^. In addition to pathological type, tumor size and the type of human papilloma virus(HPV) infection were also considered to be important causes for the radiosensitivity of cervical cancer^35, 36^.

In consideration of the poor outcomes of patients with cervical AC, more effective protocols are required for these patients. Adjuvant chemotherapy or neoadjuvant is a possible strategy. According to a Chinese clinical trial, 880 patients with FIGO stage IIB-IVA cervical AC were randomly assigned to receive only CCRT or CCRT with one cycle of neoadjuvant chemotherapy and two cycles of consolidation chemotherapy. Subsequently, patients treated by CCRT along with chemotherapy had better OS, DFS and local control after a median follow-up of 60 months. The above outcomes implicate that combined CCRT and chemotherapy is promising to enhance the survival of patients with cervical AC^37^.

The NCI-supported SEER database is the most authoritative and largest source for tumor incidence and survival. The large-scale, publicly available SEER dataset can be reliably used to guide anti-cervical AC therapy. As far as we know, our research includes the largest subjects to investigate prognostic parameters for cervical AC in the past 10 years. Inevitably, there are still several limitations in our study. Firstly, selection bias and the effects of inaccessible variables from the SEER dataset are unavoidable due to the nonrandomized nature of our research^17, 38^. Secondly, information on HPV^7, 39^, comorbidities and medication use were inaccessible from SEER database, which are considered as valuable indicators for survival of cervical cancer. Thirdly, SEER fails to provide all data to completely address our hypothesis, such as detailed information on chemotherapy and radiotherapy. Nevertheless, the currently accessible information from SEER database could fit our objectives. While the above-mentioned issues should be further addressed.

## Conclusions

Marital status, age, grade, tumor size, FIGO stage, pelvic LNM, surgery and chemotherapy were significantly associated with the prognosis of cervical AC. Patients without surgery could significantly benefit from chemotherapy and radiotherapy. Stage II–IV patients could significantly benefit from chemotherapy. In addition, only stage III patients could obtain significant survival benefit from radiotherapy. This is the largest study to investigate the clinicopathological characteristics and outcomes for patients with cervical AC. The present findings in our study are vital to the disease management and future prospective studies for this rare cancer.

## References

[1] Bray F (2018). Global cancer statistics 2018: GLOBOCAN estimates of incidence and mortality worldwide for 36 cancers in 185 countries. CA Cancer J. Clin..

[2] Siegel RL, Miller KD, Jemal A (2017). Cancer statistics, 2017. CA Cancer J. Clin..

[3] Alfsen GC, Thoresen SO, Kristensen GB, Skovlund E, Abeler VM (2000). Histopathologic subtyping of cervical adenocarcinoma reveals increasing incidence rates of endometrioid tumors in all age groups: a population based study with review of all nonsquamous cervical carcinomas in Norway from 1966 to 1970, 1976 to 1980, and 1986 to 1990. Cancer.

[4] Williams NL, Werner TL, Jarboe EA, Gaffney DK (2015). Adenocarcinoma of the cervix: should we treat it differently?. Curr. Oncol. Rep..

[5] Cracchiolo B, Kuhn T, Heller D (2016). Primary signet ring cell adenocarcinoma of the uterine cervix—a rare neoplasm that raises the question of metastasis to the cervix. Gynecol. Oncol. Rep..

[6] Mabuchi Y (2015). Clinicopathologic factors of cervical adenocarcinoma stages IB to IIB. Int. J. Gynecol. Cancer.

[7] Gadducci A, Guerrieri ME, Cosio S (2019). Adenocarcinoma of the uterine cervix: pathologic features, treatment options, clinical outcome and prognostic variables. Crit. Rev. Oncol. Hematol..

[8] Sugalski JM (2019). National comprehensive cancer network infusion efficiency workgroup study: optimizing patient flow in infusion centers. J. Oncol. Pract..

[9] Chen JL (2014). Differential clinical characteristics, treatment response and prognosis of locally advanced adenocarcinoma/adenosquamous carcinoma and squamous cell carcinoma of cervix treated with definitive radiotherapy. Acta Obstet. Gynecol. Scand..

[10] Hu K, Wang W, Liu X, Meng Q, Zhang F (2018). Comparison of treatment outcomes between squamous cell carcinoma and adenocarcinoma of cervix after definitive radiotherapy or concurrent chemoradiotherapy. Radiat. Oncol..

[11] Nosaka K (2015). Cytoplasmic maspin expression correlates with poor prognosis of patients with adenocarcinoma of the uterine cervix. Yonago Acta Med..

[12] Baalbergen A, Ewing-Graham PC, Hop WC, Struijk P, Helmerhorst TJ (2004). Prognostic factors in adenocarcinoma of the uterine cervix. Gynecol. Oncol..

[13] Park JY (2011). Outcomes after radical hysterectomy according to tumor size divided by 2-cm interval in patients with early cervical cancer. Ann. Oncol..

[14] Alfsen GC, Reed W, Sandstad B, Kristensen GB, Abeler VM (2003). The prognostic impact of cyclin dependent kinase inhibitors p21WAF1, p27Kip1, and p16INK4/MTS1 in adenocarcinomas of the uterine cervix: an immunohistochemical evaluation of expression patterns in population-based material from 142 patients with international federation of gynecology and obstetrics stage I and II adenocarcinoma. Cancer.

[15] Yu JB, Gross CP, Wilson LD, Smith BD (2009). NCI SEER public-use data: applications and limitations in oncology research. Oncology (Williston Park, N. Y.).

[16] Cahill KS, Claus EB (2011). Treatment and survival of patients with nonmalignant intracranial meningioma: results from the Surveillance, Epidemiology, and End Results Program of the National Cancer Institute. Clinical article. J. Neurosurg..

[17] Dudley RW (2015). Pediatric choroid plexus tumors: epidemiology, treatments, and outcome analysis on 202 children from the SEER database. J. Neuro-oncol..

[18] Dudley RW (2015). Pediatric low-grade ganglioglioma: epidemiology, treatments, and outcome analysis on 348 children from the surveillance, epidemiology, and end results database. Neurosurgery.

[19] Sherman ME, Wang SS, Carreon J, Devesa SS (2005). Mortality trends for cervical squamous and adenocarcinoma in the United States. Relation to incidence and survival. Cancer.

[20] Wang SS, Sherman ME, Hildesheim A, Lacey JV, Devesa S (2004). Cervical adenocarcinoma and squamous cell carcinoma incidence trends among white women and black women in the United States for 1976–2000. Cancer.

[21] Wu SG (2018). Survival in signet ring cell carcinoma varies based on primary tumor location: a Surveillance, Epidemiology, and End Results database analysis. Expert Rev. Gastroenterol. Hepatol..

[22] Chen Z (2020). Marital status independently predicts non-small cell lung cancer survival: a propensity-adjusted SEER database analysis. J. Cancer Res. Clin. Oncol..

[23] Wu SG (2018). The effect of marital status on nasopharyngeal carcinoma survival: a surveillance, epidemiology and end results study. J. Cancer.

[24] Oncology FCOG (2014). FIGO staging for carcinoma of the vulva, cervix, and corpus uteri. Int. J. Gynaecol. Obstet..

[25] Ayhan A (2019). Is the revised 2018 FIGO staging system for cervical cancer more prognostic than the 2009 FIGO staging system for women previously staged as IB disease?. Eur. J. Obstet. Gynecol. Reproduct. Biol..

[26] Yang J, Cai H, Xiao ZX, Wang H, Yang P (2019). Effect of radiotherapy on the survival of cervical cancer patients: an analysis based on SEER database. Medicine.

[27] Yuan Y, You J, Li X, Wang W (2020). Adjuvant chemotherapy after radiotherapy or concurrent chemoradiotherapy for pelvic lymph node-positive patients with locally advanced cervical cancer: a propensity score matching analysis. Int. J. Gynecol. Cancer.

[28] Rose PG (2014). Locally advanced adenocarcinoma and adenosquamous carcinomas of the cervix compared to squamous cell carcinomas of the cervix in gynecologic oncology group trials of cisplatin-based chemoradiation. Gynecol. Oncol..

[29] Katanyoo K, Sanguanrungsirikul S, Manusirivithaya S (2012). Comparison of treatment outcomes between squamous cell carcinoma and adenocarcinoma in locally advanced cervical cancer. Gynecol. Oncol..

[30] Lee YY (2011). A comparison of pure adenocarcinoma and squamous cell carcinoma of the cervix after radical hysterectomy in stage IB-IIA. Gynecol. Oncol..

[31] Wu SY, Huang EY, Lin H (2019). Optimal treatments for cervical adenocarcinoma. Am. J. Cancer Res..

[32] Bean LM, Ward KK, Plaxe SC, McHale MT (2017). Survival of women with microinvasive adenocarcinoma of the cervix is not improved by radical surgery. Am. J. Obstet. Gynecol..

[33] Yokoi E (2017). Impact of histological subtype on survival in patients with locally advanced cervical cancer that were treated with definitive radiotherapy: adenocarcinoma/adenosquamous carcinoma versus squamous cell carcinoma. J. Gynecol. Oncol..

[34] Xiong Y (2015). Combination of external beam radiotherapy and Californium (Cf)-252 neutron intracavity brachytherapy is more effective in control of cervical squamous cell carcinoma than that of cervical adenocarcinoma. Med. Oncol..

[35] Zamulaeva I (2021). Radiation response of cervical cancer stem cells is associated with pretreatment proportion of these cells and physical status of HPV DNA. Int. J. Mol. Sci..

[36] Zamulaeva IA (2020). Correlation of radiation response of cervical cancer stem cells with their initial number before treatment and molecular genetic features of papillomavirus infection. Bull. Exp. Biol. Med..

[37] Tang J, Tang Y, Yang J, Huang S (2012). Chemoradiation and adjuvant chemotherapy in advanced cervical adenocarcinoma. Gynecol. Oncol..

[38] Hankinson TC (2016). Short-term mortality following surgical procedures for the diagnosis of pediatric brain tumors: outcome analysis in 5533 children from SEER, 2004–2011. J. Neurosurg. Pediatr..

[39] Lau HY (2009). The relationship between human papillomavirus and Epstein-Barr virus infections in relation to age of patients with cervical adenocarcinoma. Taiwan J. Obstet. Gynecol..

